# “I Always Feel Like I Have to Rush…” Pet Owner and Small Animal Veterinary Surgeons’ Reflections on Time during Preventative Healthcare Consultations in the United Kingdom

**DOI:** 10.3390/vetsci5010020

**Published:** 2018-02-08

**Authors:** Zoe Belshaw, Natalie J. Robinson, Rachel S. Dean, Marnie L. Brennan

**Affiliations:** Centre for Evidence-based Veterinary Medicine, University of Nottingham, Sutton Bonington Campus, Leicestershire LE12 5RD, UK; z.belshaw.97@cantab.net (Z.B.); natalie.robinson@nottingham.ac.uk (N.J.R.); rachel.dean@nottingham.ac.uk (R.S.D.)

**Keywords:** preventative healthcare, dog, cat, veterinary, consultation, time, qualitative, animal welfare, interviews

## Abstract

Canine and feline preventative healthcare consultations can be more complex than other consultation types, but they are typically not allocated additional time in the United Kingdom (UK). Impacts of the perceived length of UK preventative healthcare consultations have not previously been described. The aim of this novel study was to provide the first qualitative description of owner and veterinary surgeon reflections on time during preventative healthcare consultations. Semi-structured telephone interviews were conducted with 14 veterinary surgeons and 15 owners about all aspects of canine and feline preventative healthcare consultations. These qualitative data were thematically analysed, and four key themes identified. This paper describes the theme relating to time and consultation length. Patient, owner, veterinary surgeon and practice variables were recalled to impact the actual, versus allocated, length of a preventative healthcare consultation. Preventative healthcare consultations involving young, old and multi-morbid animals and new veterinary surgeon-owner partnerships appear particularly susceptible to time pressures. Owners and veterinary surgeons recalled rushing and minimizing discussions to keep consultations within their allocated time. The impact of the pace, content and duration of a preventative healthcare consultation may be influential factors in consultation satisfaction. These interviews provide an important insight into the complex nature of preventative healthcare consultations and the behaviour of participants under different perceived time pressures. These data may be of interest and relevance to all stakeholders in dog and cat preventative healthcare.

## 1. Introduction

Until recently, preventative healthcare consultations for dogs and cats in the United Kingdom (UK) were assumed to have a relatively simple focus on vaccines and parasite control. However, Robinson, et al. [1,2] identified preventative healthcare consultations to be more likely to include both a full clinical examination and discussion of multiple problems than consultations for a specific health problem. The median length of preventative healthcare consultations at two UK practices involved in that research was 9 min and 45 s with an interquartile range of 7 min and 50 s to 14 min [3]. Concerns have been raised about whether UK small animal consultation lengths, particularly preventative healthcare consultations, are adequate [3,4]. However, there are no data reporting UK owner or veterinary surgeon attitudes towards consultation lengths or any consequences of the time allocation being too short. Similarly, it has not been previously established whether preventative healthcare appointments are under time pressure as a result of their complexity, or whether this might lead to tensions related to a need to keep to time.

Literature on the impact of consultation lengths does exist in the human medical field. Doctors report that short consultation lengths can result in an impaired ability to implement preventative healthcare and health promotions [5], a reduction in health screening [6] and being forced to prioritise certain health problems over others during a consultation [7]. Patients who felt their consultation was too short reported less likelihood of compliance with the recommendations made [8]. It is unknown whether these factors are significant in small animal veterinary care.

The aim of this study was to describe owner and veterinary surgeon perspectives about consultation lengths and time in relation to preventative healthcare consultations using a qualitative methodology. The objective was to perform semi-structured interviews to discuss recent owner and veterinary surgeon experiences of preventative healthcare consultations and to identify key emergent themes using thematic analysis.

## 2. Materials and Methods

This study forms part of a larger body of work, the aims of which were to ascertain what the expectations, opinions and experiences of veterinary surgeons and owners were in relation to preventive medicine consultations in the UK, and to understand their perspectives on what could be done to maximise the benefits of preventive medicine consultations. Data were gathered through telephone interviews during July and August 2016 with dog and cat owners and veterinary surgeons conducting small animal consultations in the UK. The study was conducted in accordance with the Declaration of Helsinki and ethical approval was granted by the ethics committee at the School of Veterinary Medicine and Science, University of Nottingham (Reference number: 1521 150813). Reporting follows the Consolidated Criteria for Reporting Qualitative Research (COREQ) checklist [9].

### 2.1. Owner and Veterinary Surgeon Recruitment

Recruitment for interview was based on separate purposive sampling frames designed by the authors NR and RD (available on request) [10]. Purposive sampling involves identifying and selecting interviewees knowledgeable about the topic of interest and likely to represent a broad range of experiences [10]. Sampling frames designed by Everitt [11] and Belshaw [12] were used for reference. The owner sampling frame included owner, pet and practice variables; the veterinary surgeon sampling frame included veterinary surgeon and practice variables.

Owner interviewees were included if they met the following two criteria: (a) ownership of one or more dog and/cat that had attended a preventative healthcare consultation in the UK during the preceding 3 months; and (b) willingness to be interviewed by telephone about that consultation during the data collection period. Eligible preventative healthcare consultations were: vaccinations; titre testing; parasite control; health check; or prevention of season. Veterinary surgeons were included if they met the following three criteria: (a) those currently working in a general practice in the UK; and (b) who regularly performed preventative healthcare consultations; and (c) who were available for interview during the data collection period.

Owners were recruited via: direct approach of owners within the authors’ networks; social media posts to Facebook and Twitter; recruitment of eligible owners by veterinary surgeons in a Scottish multi-branch veterinary practice; and snowball sampling, whereby recruited participants help to identify others [10]. Veterinary surgeon recruitment was conducted by NR using: Twitter and Facebook posts; by contacting veterinary practices who had expressed an interest in collaboration with the Centre for Evidence-based Veterinary Medicine; direct emails to the list of small animal practices on the Royal College of Veterinary Surgeons’ database; and snowball sampling. 

### 2.2. Interview Procedure

Owners and veterinary surgeons who expressed an interest in being interviewed were emailed information and a copy of the consent form. Those willing to participate were asked to supply information relevant to the inclusion criteria to confirm their eligibility prior to an interview date being arranged, and demographic details were collected at this point. Incentives to participate were not provided. All telephone interviews were conducted by NR, a veterinary surgeon who had received qualitative research training. Prior to each interview, the consent form was read to each participant and verbal consent to proceed was granted. Separate semi-structured interview guides [13] were used for owner and veterinary surgeon interviews. These guides had been pilot tested with two owners and two veterinary surgeons; minor changes were made to the question order before data collection. To minimize recall bias, owners were asked to focus their discussion on the preventative healthcare consultation(s) that they had attended during the preceding 3 months, whilst dog and cat preventative healthcare consultations in general were discussed with veterinary surgeons. Relevant to this publication, if they had not mentioned it themselves, owners and veterinary surgeons were asked near the end of the interview whether they felt the length of their recent consultation had been adequate and what they might discuss or do given more time.

### 2.3. Data Analysis

Interviews were recorded using a Dictaphone and telephone adapter before being professionally transcribed verbatim. Transcripts were not returned to interviewees and repeat interviews were not performed. Data analysis was performed by ZB, a veterinary surgeon with training and previous experience in conducting qualitative research. Each transcript was checked for accuracy against the audio recordings and any discrepancies amended. Thematic analysis was then performed following the six-step plan as described by Braun and Clarke [14] using the organisational support of nVivo (nVivo qualitative data analysis software v11, QSR International Pty Ltd., Melbourne, Australia). These six steps are summarised as: (1) reading through the entire dataset to become familiar with its content; (2) generation of initial “codes” of interest, where a code is defined by Braun and Clarke as “the most basic segment, or element, of the raw data which can be assessed in a meaningful way regarding the phenomenon”; (3) searching for “themes”, a descriptive term for a pattern within the data which captures something relevant to the research question; (4) reviewing candidate themes to ensure they form a coherent pattern, are valid, and that all relevant codes can be described; (5) defining, refining and naming key themes that capture data important to the research question; and (6) writing a report to describe each theme and the merit of the analysis performed.

Codes that summarised the meaning of extracts of the transcribed text were inductively identified by ZB through iterative reading of transcripts. All transcripts were completely coded; many extracts were coded multiple times as sentences frequently contained content relevant to multiple codes. As new codes were developed, transcripts already coded were re-checked to determine whether extracts fitted the new codes. Codes were then inductively grouped according to their content to form subthemes. Subthemes were iteratively reviewed and re-organised to identify the most comprehensive way of presenting the data. Content of the codes was regularly re-checked to ensure the meaning of the extracts was compatible with the subtheme. Subthemes were mapped onto a single large sheet of paper and manually organised into inductive and deductive themes containing related subthemes. Double coding was not performed for reasons described by Morse [15], but themes were repeatedly reviewed and re-organised by ZB and MB as their content was progressively refined through the writing of each theme.

Importantly, in contrast to other qualitative methods such as content analysis, key themes in thematic analysis are not determined in a quantifiable manner as they represent the most important, not necessarily the most prevalent themes, identified in the dataset [14]. Statistical analysis was not performed as is standard in qualitative research [16,17]. Data saturation was reached when no additional themes emerged during as a result of analysing further transcripts.

## 3. Results

Thirty-one interviews were arranged, but two owners were unable to participate due to unforeseen circumstances. Twenty-nine telephone interviews were conducted with 15 owners and 14 veterinary surgeons. Interviews lasted 15 to 59 min (median 28 min; interquartile range 21–40.5 min). Demographic details of the veterinary surgeons, the veterinary practices, the owners and their pets are provided in supplementary materials (see Tables S1 and S2). The ten female and four male veterinary surgeons were from 12 veterinary practices across the UK. Two practices treated animals of all species, the remaining 10 treated only small animals. All veterinary surgeons had graduated in the preceding 20 years but covered a range of junior to senior practice roles. Preventative healthcare consultations in those practices ranged from 10–20 min in length. The 15 pet owners owned 19 dogs and 3 cats which ranged from 6 months to 11 years in age; all owners interviewed were female as male owners were very difficult to recruit.

Thematic analysis deductively identified four key themes, each of which has been reported separately. These described: motivators and barriers to using preventative medicines [18]; expectations of owners and veterinary surgeons about what would happen during preventative healthcare consultations [13]; the role of veterinary nurses and receptionists in preventative healthcare [19]; and the importance of the length of preventative healthcare consultations. This last key theme is reported below with illustrative quotes.

### 3.1. Owners

Time was discussed by owners in terms of the number of minutes the preventative consultation lasted, how the time was used, and whether they felt that they had been under time pressure. Some owners were unsure how long their allocated consultation length had been. Where specific lengths were recalled, these were typically 10–15 min, but one owner described a 30 min consultation for her pets’ antibody titre test. Most owners felt that the length of time they had been in the consulting room had been what they had expected, whilst others were aware the veterinary surgeon had extended the consultation length beyond the time allocated.

“If we need to be in there longer than the appointment slot, then we’re in there longer than the appointment slot. We’re never rushed. If we need to discuss something, we can discuss it in detail. We never feel rushed even if it means that she’ll run behind a little bit.”

[Owner 6]

However, several owners identified that different veterinary surgeons took different lengths of time over preventative healthcare consultations, and that sometimes a preventative healthcare consultation was shorter than the time that they thought had been allocated. Some felt this was because the veterinary surgeon was not interested in them or their pet, whilst others considered it to be a response to the pressure of having a full waiting room.

“I feel like the vet was very pressurised to get through the people. Sort of to get through us to go on to the next people.”

[Owner 7]

The pace of the consultation appeared to be at least as important as its length. Consistently, owners discussed whether they thought the consultation had felt “rushed”. Most considered they had been given all the time that they needed without feeling rushed, and many identified the communication style or personality of the veterinary surgeon to be important in this. However, a few felt that their recent preventative healthcare consultation had been conducted at a faster pace than they would have liked, with the veterinary surgeon either skipping elements of the clinical examination or spending inadequate time on discussions. Owners appeared not to feel they could do anything about being rushed during that consultation.

“It was really quick this one, you know. Erm, he didn’t do anything… considering he was a ten-week old puppy, I was really disappointed… Disappointed and annoyed with myself when I came out that I hadn’t said…hadn’t said to him, whoa, hang on. Can you please listen to his heart? Can you please check him over?”

[Owner 10]

Conversely, some owners felt that they needed to rush the consultation themselves. Typically, this was described to be motivated by the perception that the veterinary surgeon was busy and knowledge that there were a lot of others in the waiting room.

“I think I always feel like I have to rush, because I know what it feels like to have a time constraint. I don’t want to keep them up and I just want to get in and out.”

[Owner 1]

The sense of whether the consultation had been rushed by either veterinary surgeon or owner appeared to impact owner satisfaction. Owners who said they were broadly happy with their recent consultation typically described it to have involved veterinary surgeons who took time over greeting the owner and their pet, performing a thorough clinical examination and answering any questions that the owner raised without making them feel time pressured.

“I think there’s a lad there who’s a…I think he’s a trainee. But he’s been there a couple of years now and he’s quite good when we get him cos he tends to take his time and go through everything so… but you’ll get other more experienced vets that are quite quick in and out sort of thing so…yeah. I prefer seeing the trainee lad, I think, cos he stops, listens and you can see he’s taking his time over everything…”

[Owner 9]

Owners who described their consultation as rushed typically described the clinical examination and/or discussion of problems identified had been given inadequate time. Those owners consistently identified topics that they would have liked to discuss or questions that they forgot to ask. Consequently, discussions did not occur during rushed consultations about aspects of their pets’ health that these owners felt were important.

“I think, you know, [the vet] was just thinking “Oh this is an easy one”—a quick vaccination and that’s it, which I can fully understand, I mean [dog’s name] is fine but she is a Westie dog and we do have little issues with her and I think it’s nice to relax a bit and talk to the vet about those things…. She’s got some behavioural problems and I would have discussed that a little bit, I think”

[Owner 15]

Having adequate time for discussion appeared particularly important when new health problems were identified by the veterinary surgeon. Several owners expressed frustration that insufficient time had been given to asking their own perspectives about a problem, or to fully explaining its implications for them and their pet.

“And maybe if she’d had more time, maybe she would have been…asked more questions about her weight rather than just kind of like pointing the finger and saying, she’s overweight. I felt like it should have been a little bit more, kind of like taking note of the…I’m not lying, I am telling you that I’m aware of it.”

[Owner 7]

In contrast, owners who did not feel that their consultation had been time-pressured recalled a discussion that had been valuable to them because the veterinary surgeon had fully explained the problem, solicited their opinion and helped them to make a clear plan of action.

“I took him in because his vaccination was due so we went along to the vet and whilst I was there having his injection and I got into trouble because he was overweight…. I’ll be honest [the consultation] was longer than I thought it would be, but I felt it needed to be so I could fully understand how much to feed him, when to feed him all of those kind of things. She took the time to make sure I understood everything before I left the surgery.”

[Owner 11]

Owners discussed several consequences of consultations that they felt were too short for any reason, or had felt rushed. These included: specifically avoiding those veterinary surgeons or practices; using alternative information sources such as the internet or receptionists after the consultation to answer additional questions; or soliciting the advice of another veterinary surgeon to go through unanswered questions.

“Because he is a labradoodle we’re members of various different doodle groups, so all these groups all have this type of dog. So I would ask in those groups, because you’ve got all owners of the same breed, so they’ve got… they’ll have similar experiences and they might know the answer to a question I’ve got.”

[Owner 2]

Owners also made suggestions for changes that could be made in veterinary practices to mitigate time pressures. These included: increasing the length of consultations; that veterinary surgeons did not try to discuss new health problems during preventative healthcare consultations; including time with a veterinary nurse after a consultation during which any additional questions could be asked; and inviting owners to spend time prior to the consultation compiling a list of questions they wanted to ask so that these could be prioritised.

“It’s a busy practice and when you go you’re conscious that there’s people waiting to follow you and things...so it would be better if you were allotted a bit more time and even if it was with the veterinary nurse. That would be just as good for general stuff.”

[Owner 15]

“I think if there was a questionnaire with sort of maybe some ticky boxes: weight, food, you know, a list of things you could tick off that you might want to discuss, it might help you remember what you…you know, or it might make you think, oh actually, I haven’t spoken about the food or how the weight’s going or fleaing whatever treatment.”

[Owner 6]

### 3.2. Veterinary Surgeons

All veterinary surgeons discussed the challenges of trying to keep to time, and the impact of not having adequate allocated time for some preventative healthcare consultations. Most worked in practices where initial puppy or kitten vaccination consultations were allocated 20 min and all subsequent preventative healthcare consultations were given 10 min; a few had 15 min for all consultation types. Many discussed the need to be flexible with consultation lengths across each consulting session; some consultations would require longer than the allotted time, so others were then effectively shortened to catch up. Preventative healthcare consultations could both add to, and be used to mitigate, time pressures. Consistently, veterinary surgeons suggested preventative healthcare consultations involving very young, old or multi-morbid pets were likely to exceed their allotted 10–15 min in length as there were multiple different problems to discuss. This appeared to be driven by an almost universal agreement that every health-related problem identified during the clinical examination should be discussed during the preventative healthcare consultation rather than being deferred to a subsequent consultation.

“I think animals between the age of one and eight you could do that in ten minutes, there’s generally not a lot involved but I think once you get to the older animals they do tend to take a bit longer because you pick up more problems really.”

[Veterinary surgeon 4]

A few veterinary surgeons described consistently consulting through designated break times to make up lost time whilst others described consciously changing their communication style during some preventative healthcare consultations to minimise the length of any discussions. Consistently, a busy waiting room appeared to increase the sense of time pressure. Several implied that preventative healthcare consultations for young, healthy adults were often used to catch up on time.

“As I say it just depends, it depend on the day. If I am busy and we’ve got loads of people waiting then I am afraid they get a history, clinical exam and you know check everything is okay, are you worried about anything. If not, if I am quiet and I have loads of time to go through everything properly. So it does depend on time.”

[Veterinary surgeon 11]

Those who worked in branch clinics typically felt that they had more ability to adjust their allocated consultation lengths, both because they were able to use their knowledge of their clients to predict which consultations would need more time, and because they perceived that they usually had fewer consultations.

“I don’t have a full day so there is opportunity for me to come in the morning and think ‘This is a cat I’ve seen very, very frequently, it’s very old, I’ll probably extend that one by five minutes’. There are standard lengths but if I look at something and think that needs a little bit longer then I’ll just alter it on the timetable because I’ve got freedom to alter things.”

[Veterinary surgeon 2]

Time budgeting was challenging, and some suggested that trying to manage time required conscious attention during some consultations. Owners who did not appear interested in any discussion of their pets’ health were contrasted with those who sought in-depth discussion of a range of topics.

“I mean some people definitely don’t want to go into much detail, you know you get the impression that they just want it vaccinated and to leave. But for those people that want more information then you know, if you are trying to fit it into 10 minutes you are just overrun. It’s impossible to fit everything in.”

[Veterinary surgeon 12]

Veterinary surgeons’ attitudes towards educating owners during a preventative healthcare consultation appeared to range from those happy just to deal with immediate problems to those keen to ensure that the owner fully understood what they should be doing to keep their pet healthy. As a result, 10 min was adequate for most preventative healthcare consultations for some veterinary surgeons, but was never long enough for others who felt that significantly more content needed to be included in each consultation.

“I suppose there’s some vets in my practice that will happily whizz through ten minute appointments but their vaccination booster will be quickly checking over and then jab it and it’s out the door probably within six or seven minutes whereas mine I go into all the extra things. I chat about doing the dental and doing the joint care and I’m constantly bringing things up … I don’t think ten minutes is enough at all.”

[Veterinary surgeon 3]

Almost all veterinary surgeons listed topics that they would like to discuss in more depth or procedures that they would be able to do if they had longer preventative healthcare consultations. Most suggestions were focused on an enhanced ability to screen for, and explain, diseases of old age. Several identified that longer consultations would provide them with a better opportunity to educate owners about other aspects of pet health such as diet or behaviour. Some thought longer consultations, particularly for older animals, would enhance compliance with recommendations as more time would be available both to explain and to build rapport. However, clients’ unwillingness to pay for longer consultations and the variation in apparent client interest in pet health were identified as barriers to practices lengthening all preventative healthcare consultations.

“If it was a bit stiff and sore, I’d take it out into the car park and walk it up and down and go oh yes, he is a bit lame on that right paw. 10 year old Westie who has got a sore elbow, you know I would do that. I would do more and I would talk more at length about things. If people were interested. I mean people aren’t often interested at that time.”

[Veterinary surgeon 11]

“Some people just want the booster they don’t want anything else, they’re quite happy but some of that is understanding related and how much time they’ve given themselves for the appointment on the day. I don’t think people want to pay any more than they’re currently paying for what they’re getting…”

[Veterinary surgeon 5]

## 4. Discussion

Reflections on the impact of time formed a large part of our interview data about preventative healthcare consultations. These data describe a complex relationship between allocated consultation length, actual consultation length, consultation content and consultation pace, with different individuals placing different value on each of those metrics. Owner, veterinary surgeon and practice factors have been identified as contributors to actual, versus allocated, consultation lengths. Description of the negative consequences of perceived time constraints on satisfaction and consultation content is novel and important, as is the finding that owners and veterinary surgeons modify their behaviour to mitigate perceived time pressures. Time management by veterinary surgeons during consultations has been revealed to be a conscious, complex, challenging task which should be recognised by employers and those involved in veterinary education. We recommend that interventions designed to improve any outcome in relation to preventative healthcare consultations must take these factors into account.

Given the previous work that has identified preventative healthcare consultations to be particularly complex yet allocated no additional time [1,2,3,4], it is unsurprising that these interviews identified a theme relating to time pressures. Scoring videos of small animal consultations recorded in Canada, Shaw, et al. [20] rated veterinary surgeons being “rushed” or “hurried” during 20% of problem appointments and 13% of wellness appointments. Our study suggests this phenomenon may be very common in UK preventative healthcare consultations. Combined with our novel findings that owners may also feel compelled to rush, and that both owners and veterinary surgeons may be deliberately avoiding certain topics during preventative healthcare consultations to save time, this is extremely concerning.

Robinson [1,2] previously identified that preventative healthcare consultations are more likely than other consultation types to include discussion of multiple problems. The veterinary surgeon interviewees suggest multi-problem preventative healthcare consultations, particularly those involving young, old or ill patients, may be most at risk of time pressures. Parallels exist in human healthcare. Increasing patient age has been reported as a factor associated with discussion of a greater number of problems per consultation in general practice [21] and video recordings of consultations [7] identified that doctors also discuss multiple problems within a single consultation under great time pressure. Negative impacts of short discussions about several problems during a single consultation has led to recommendations that the standard consultation length of patients with multiple comorbidities should be increased in medical practice [22,23]. Our interviews suggest that similar consideration should be given to increasing the length of preventative healthcare consultations in pets with known pre-existing health problems or in populations (based on age, breed or both) that may be more likely to have such problems. Quantitative surveillance data from groups such as VetCompass [24,25] may be useful in predicting which animals these are likely to be. We recognise that any proposed change to the length of consultations will be met with concerns about logistics, supply, demand and profit. Case studies which address these challenges from practices who have already altered the allocated length of consultations will be helpful.

However, simply increasing consultation times alone may not be sufficient. Previous research, [3,4] has identified that consultations in the UK rarely take the actual length of time allocated to them. Our interviews further substantiate this. Additionally, our research has identified that judgements of “enough” time appear to be very complex. These interviews demonstrate the need to consider not only time allocation for veterinary consultations, but how that time is used and how the sense of time makes both participants in the consultation feel. Owners discussed time in terms of minutes they thought they should have been in the consultation room; the number of minutes they thought the consultation had lasted; how well they felt that time was used by the veterinary surgeon; whether the pace of the consultation was as they expected; and whether they felt they needed to alter that pace. Veterinary surgeons additionally discussed time in relation to the other consultations they had to fit into that consultation session. Future research on the theme of consultation lengths, particularly in relation to consultation satisfaction, must reflect this complexity.

Control over consultation length has been identified as important for doctors’ job satisfaction [26]; these interviews suggest the same may be important to veterinary surgeons. Whilst Shaw, et al. [27] did not identify a relationship between the length of Canadian veterinary consultations and the veterinary surgeons’ satisfaction with the consultation, the current study suggests both veterinary surgeons and owners may feel dissatisfaction as a result of rushed consultations. This is again supported by research in the medical field. Patients visiting their medical general practitioner who thought their consultation felt too short were more dissatisfied, independent of the actual length of that consultation [8]. Grave and Tanem [28] identified a similar phenomenon manifesting in reduced compliance in owners of dogs prescribed short-term antibiotics who thought their consultation was too short. Criticism has been levelled at owners for using alternative information sources such as the internet [29], but the current study suggests that feeling there was inadequate time for discussion during a preventative healthcare consultation may be at least in part to blame. Given the poor quality of online pet health information [30,31,32], this is potentially a serious problem.

This study has some limitations. Male owners and cat owners were particularly difficult to recruit, with other aspects of both sampling frames addressed. Owners were recruited predominantly from the north of England. As little data exist comparing any aspect of consultations by region in the UK, the impact of this on how widely these data can be extrapolated is unknown. Given the qualitative and recall-based nature of these data, it is not possible to verify how accurately these interviews reflect what actually happened in the consulting room. Owners’ recall of both how long consultations were allocated and how long they were in the room may be inaccurate, and veterinary surgeons may have described outlying consultations. Ascertaining when data saturation has been reached during qualitative research is challenging [15], particularly when the interview subjects are relatively wide-ranging as in this instance but our thematic analysis suggested we had recruited sufficient interviewees. Discussions of the nature of time were complex, and it was not always easy to distinguish whether participants were referring to the pace of a consultation or the length of time over which it was conducted. Despite these limitations, since such little information is available about the impact of time on veterinary consultations, this study provides an important step forward. Our data concur well with other publications about UK dog and cat consultations [1,2,3,4], and human behaviour during medical encounters [7,8], suggesting that recall bias did not adversely affect our data. Video-recorded preventative healthcare consultations, coupled with contemporaneous interviews with participants, would permit further exploration of the relationship between time and consultation satisfaction that is suggested by these interviews.

## 5. Conclusions

This publication is the first description of veterinary surgeons’ and owners’ perspectives of time in relation to dog and cat preventative healthcare consultations. Through using a qualitative methodology, novel to this field, we revealed that a complex relationship exists between allocated versus actual consultation length, the content of the consultation and the pace at which the consultation is conducted. These data demonstrate that allocated consultation length may be a poor measure of the actual length of each consultation, or of the quality of that consultation as perceived by veterinary surgeons and owners. Patient, owner, veterinary surgeon and practice factors have been identified as variables that may impact the actual length of a preventative healthcare consultation. Determining the effect of different allocated, and delivered, consultation lengths on client satisfaction, veterinary surgeon wellbeing and job satisfaction, and the impact of the consultation on patient welfare, will be important future work. 

## Supplementary Materials

The following are available online at http://www.mdpi.com/2306-7381/5/1/20/s1, Table S1: Demographic details of owners interviewed and their pets. Table S2: Demographic details of veterinary surgeons interviewed and their practices.
