# Supplementary material for: “I Always Feel Like I Have to Rush…” Pet Owner and Small Animal Veterinary Surgeons’ Reflections on Time during Preventative Healthcare Consultations in the United Kingdom
Source: Vet Sci. 2018 Feb 8;5(1):20. doi: 10.3390/vetsci5010020 (PMC5876559; doi:10.3390/vetsci5010020)
Supplement: Supplementary file 1 [file vetsci-05-00020-s001.pdf]

veterinary sciences

MDPI

Article

"I Always Feel Like I Have to Rush…" Pet Owner and Small Animal Veterinary Surgeons' Reflections on Time during Preventative Healthcare Consultations in the United Kingdom

Supplementary materials:

Table S1. Demographic details of owners interviewed, and their pets. Table reproduced from [13].

Owner ID	Sex	Owner employment status and job type	Owner location	Owned pets previously?	Veterinary practice type attended	Demographics details of pets currently owned
1	Female	Working, healthcare	North England	No	Independent	4 year old male neutered Lhasa Apso dog
2	Female	Working, undisclosed non-science job	Scotland	No	Independent	2 year old female neutered golden-doodle dog
3	Female	Working, animal care	North England	Yes	Independent	3 year old female neutered dachshund dog<br>3 year old female neutered poodle cross dog
4	Female	Working, undisclosed non-science based job	North England	Yes	Independent	3 year old female neutered domestic shorthaired rescue cat
5	Female	Working, scientist	Midlands	Yes	Independent	11 year old male entire working cocker spaniel gundog<br>6 year old female entire working cocker spaniel gundog<br>2 year old female entire working cocker spaniel gundog
6	Female	Working, undisclosed non-science job	North England	Yes	Independent	8 year old female neutered Bedlington terrier rescue dog
7	Female	Working, customer service	North England	Yes	Independent	8 year old female neutered golden-doodle dog<br>3 year old female neutered golden-doodle dog<br>9 year old female entire toy poodle dog
8	Female	Working, undisclosed non-science job	Midlands	Yes	Independent	8 year old male neutered cockerpoo agility dog<br>7 year old female neutered cockerpoo agility dog
9	Female	Working, undisclosed non-science job	North England	No	Independent	6 year old female neutered domestic shorthaired cat<br>5 year old male neutered domestic longhaired cat<br>3 year old male neutered Chihuahua dog
10	Female	Retired	North England	Yes	Independent	4 month old male entire cockerpoo dog
11	Female	Working, undisclosed non-science job	North England	Yes	Independent	7 year old male neutered cavalier king Charles spaniel dog
12	Female	Working, child care	North England	No	Corporate	6 month old female entire working cocker spaniel dog
13	Female	Retired	North England	Yes	Independent	2 year old male neutered lurcher dog
14	Female	Working, animal care	North England	No	Corporate	5 year old male neutered labradoodle dog<br>4 year old male neutered labradoodle dog
15	Female	Retired	North England	Yes	Corporate	2 year old female neutered crossbreed dog<br>10 year old male neutered west highland white terrier dog

Table S2. Demographic detail of the veterinary surgeons interviewed and their practices Table reproduced from [13].

Veterinary surgeon ID	Sex	Year of graduation	Country where veterinary education undertaken	Position within practice	Practice location	Practice description	Standard preventative medicine consultation length (minutes)
1	Female	2004	UK	Senior vet	North England	Small animal, independent, multi-branch	15
2	Male	2015	UK	Assistant	North England	Small animal, independent, multi-branch	15
3	Female	2008	UK	Assistant	Wales	Small animal, independent, multi-branch	10
4	Female	2003	UK	Assistant	South East England	Mixed, independent, multi-branch	10–20
5	Female	2000	UK	Clinical director	South England	Small animal, corporate, multi-branch	15
6	Female	2001	UK	Assistant	South East England	Small animal, corporate, multi-branch	15–20
7	Male	2006	Hungary	Clinical director	South East England	Small animal, corporate, single branch	15
8	Male	1988	UK	Partner	Midlands	Small animal, independent, single branch	10
9	Female	2003	USA	Clinical Director	Midlands	Small animal, corporate, single branch	20
10	Female	2004	UK	Assistant	Scotland	Small animal, independent, multi-branch	15
11	Female	2005	UK	Assistant	North England	Small animal, independent, multi-branch	10
12	Male	2008	UK	Assistant	North England	Mixed, independent, single branch	10
13	Female	2008	Ireland	Assistant	Scotland	Small animal, independent, multi-branch	15
14	Female	1993	UK	Assistant	South West England	Small animal, independent, multi-branch	15

© 2018 by the authors. Submitted for possible open access publication under the terms and conditions of the Creative Commons Attribution (CC BY) license (http://creativecommons.org/licenses/by/4.0/).
